# Prevalence of resistance-associated viral variants to the HIV-specific broadly neutralising antibody 10-1074 in a UK bNAb-naïve population

**DOI:** 10.3389/fimmu.2024.1352123

**Published:** 2024-03-18

**Authors:** Panagiota Zacharopoulou, Ming Lee, Thiago Oliveira, John Thornhill, Nicola Robinson, Helen Brown, Sabine Kinloch, Philip Goulder, Julie Fox, Sarah Fidler, M. Azim Ansari, John Frater

**Affiliations:** ^1^ Nuffield Department of Medicine, University of Oxford, Oxford, United Kingdom; ^2^ Department of Infectious Disease, Imperial College London, London, United Kingdom; ^3^ Laboratory of Molecular Immunology, Rockefeller University, New York, NY, United States; ^4^ Institute of Immunity and Transplantation, Royal Free Hospital, London, United Kingdom; ^5^ Department of Paediatrics, University of Oxford, Oxford, United Kingdom; ^6^ Department of Infection, Guys and St Thomas’ NHS Trust, London, United Kingdom; ^7^ Oxford National Institute of Health Biomedical Research Centre, Oxford, United Kingdom

**Keywords:** HIV - human immunodeficiency virus, primary HIV infection (PHI), broadly neutralising antibodies, 10-1074, resistance screening

## Abstract

Broadly neutralising antibodies (bNAbs) targeting HIV show promise for both prevention of infection and treatment. Among these, 10-1074 has shown potential in neutralising a wide range of HIV strains. However, resistant viruses may limit the clinical efficacy of 10-1074. The prevalence of both *de novo* and emergent 10-1074 resistance will determine its use at a population level both to protect against HIV transmission and as an option for treatment. To help understand this further, we report the prevalence of pre-existing mutations associated with 10-1074 resistance in a bNAb-naive population of 157 individuals presenting to UK HIV centres with primary HIV infection, predominantly B clade, receiving antiretroviral treatment. Single genome analysis of HIV proviral envelope sequences showed that 29% of participants’ viruses tested had at least one sequence with 10-1074 resistance-associated mutations. Mutations interfering with the glycan binding site at HIV Env position 332 accounted for 95% of all observed mutations. Subsequent analysis of a larger historic dataset of 2425 B-clade envelope sequences sampled from 1983 to 2019 revealed an increase of these mutations within the population over time. Clinical studies have shown that the presence of pre-existing bNAb mutations may predict diminished therapeutic effectiveness of 10-1074. Therefore, we emphasise the importance of screening for these mutations before initiating 10-1074 therapy, and to consider the implications of pre-existing resistance when designing prevention strategies.

## Introduction

Broadly neutralising antibodies (bNAbs) may emerge during HIV infection in a rare subset of people living with HIV (PWH). Unlike early strain-specific neutralising antibodies, bNAbs are capable of neutralising a broader range of HIV by targeting conserved regions of the HIV Envelope protein (1). Passive administration of bNAbs can sustain viral suppression in the absence of antiretroviral therapy (ART) and may be efficacious for HIV prevention (2–7). Pharmacokinetic data suggest the longer acting M428L/N434S (‘LS’) variants may provide therapeutic levels for months (2, 8–11).

The presence of bNAb-resistant proviruses integrated into latently infected cells prior to bNAb exposure, however, could compromise bNAb efficacy (2, 8, 10, 12). This may pose a risk of bNAb treatment failure not only for individuals with HIV but more widely, due to the potential for transmission of bNAb-resistant strains, as documented for ART drug resistance (13). One difficulty lies in the lack of a gold standard test for determining susceptibility to bNAbs, with approaches divided between either *in-vitro* neutralisation assays using amplified envelope (*env)* sequences or sequence-based *in-silico* predictions (14–16).

One of the most potent bNAbs in clinical trials is 10-1074, which neutralises HIV by targeting the base of the V3 loop on HIV Env (4). 10-1074 is able to interact with the glycan attached to the potential N-glycosylation site (PNG) at position 332 of HIV Env (documented position is relative to the HXB2 strain) while penetrating through the glycan shield and binding to the underlying protein region (17, 18). These traits of 10-1074, which are also associated with other V3-targeting bNAbs, are a result of extensive affinity maturation during multiple rounds of host-induced selection pressure. Either on its own or in combination with other bNAbs, 10-1074 has shown extended viral suppression for up to 21 weeks (2, 4, 8) and it is, therefore, one of the more promising agents in the field of HIV therapeutics. When tested in a panel of 118 strains, 10-1074 was very potent, although with a reported breadth of 63-67% (19). Successful roll-out of 10-1074 – and bNAbs more generally – as widely used therapeutics will be dependent on understanding the degree of bNAb resistance that exists in the general population, especially as responses are likely to be HIV subtype-specific (for example,10-1074 is reported to be more potent against B-clade viruses (17) and less potent on C and A clades (18).

This study reports the prevalence of mutations in HIV *Env* that have been associated with resistance to 10-1074 in a UK cohort of people with treated primary HIV infection (PHI). Through this analysis, we aim to understand the current prevalence of resistance to 10-1074 and map how this evolves in the population or within-host in people with HIV who have not previously been treated with 10-1074.

## Results

### HEATHER cohort demographics

**Figure 1:**
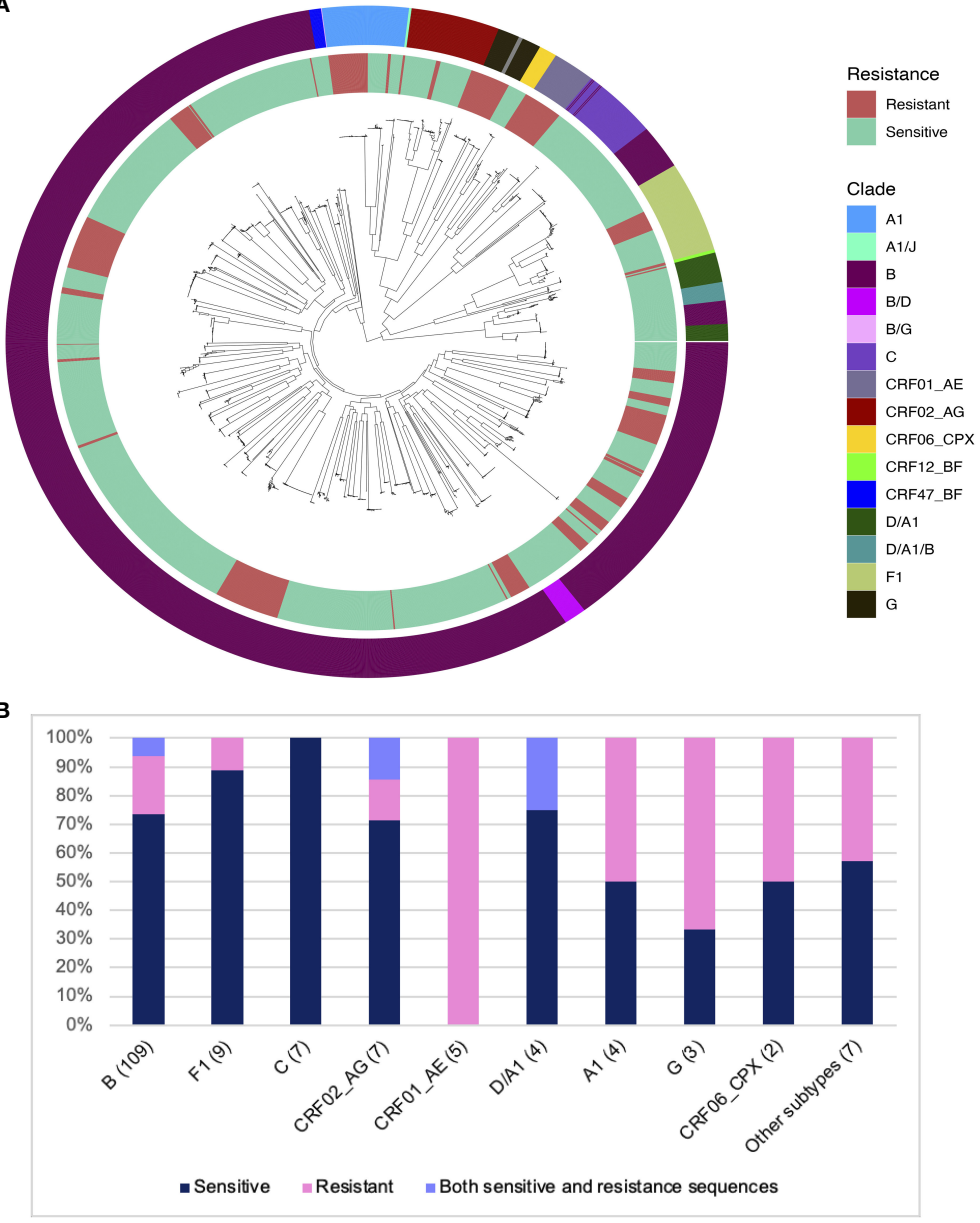
Distribution of predicted 10-1074 resistance in different HIV clades in the HEATHER cohort. **(A)** ML phylogenetic tree showing all sequences from 157 participants in the HEATHER cohort. The inner ring layer marks sequences with mutations associated with 10-1074 resistance in red and sensitive sequences in green. The outer ring layer shows the sequence clades. **(B)** Barplot showing the distribution of samples with 10-1074 resistance-conferring mutations per clade. The number in brackets is the number of samples. The percentage of samples in each clade is on the y-axis.

The HEATHER cohort was an observational study looking at the impact of ART on the viral reservoir in people with Primary HIV Infection (PHI) who started ART within 3 months of diagnosis (20). Samples from 157 participants were used to study the prevalence of resistance to 10-1074 based on sequence analysis of HIV *env*. All participants were on ART at the time of sampling for an average of 5 years (range: 0-18 years). The majority of participants (64.4%) seroconverted between 2013 and 2016 (range: 2001-2020). The median age of the participants at the time of ART initiation was 35 years (range: 18-64). All but two participants in this cohort were male (98.7%), and 91 of 92 recorded transmission events were men having sex with men (MSM). An average of 16 single, proviral *env* sequences per participant (range: 1-72 sequences) was assessed for sensitivity to bNAbs. In total, 2593 sequences were analysed in this study. Most participants had B clade HIV (69.4%), followed by F (5.7%), C (4.4%), CRF02-AG (4.4%), CRF01-AE (3.1%), A1 and D/A1 (2.5% each), with other clades representing less than 2% of the cohort (**Figure 1A**).

### Predicted 10-1074 sensitivity in the HEATHER cohort

The key determinants of HIV sensitivity to 10-1074 are the presence of a sequon at amino acid positions 332-334 of the HIV Envelope protein (a sequence of N-x-T/S, where x can be anything but P), which allows glycan attachment on the asparagine, and an intact motif on the protein,^324^G(D/N)IR^327^, which allows CDRH3 binding (4, 17). 28.6% of the HEATHER cohort participants had at least one sequence with mutations associated with 10-1074 resistance. No participants had previously been exposed to 10-1074 or had received treatment with bNAbs. Co-existence of sensitive and resistant sequences to 10-1074 in the same person was detected in 5.7% of participants in the HEATHER cohort. For this group with mixed sequences, the proportion of resistant sequences varied between12.5 and 85.7%. Subtype was an important determinant of resistance - most participants with clade B HIV in this cohort were predicted to be sensitive to 10-1074 (81/109; 74.3%). The highest rate of predicted 10-1074 resistance in the cohort was detected in CRF01-AE, although this was only five participants (5/5; 100%) (**Figure 1B**).

### Distribution of resistance-conferring variants

**Figure 2:**
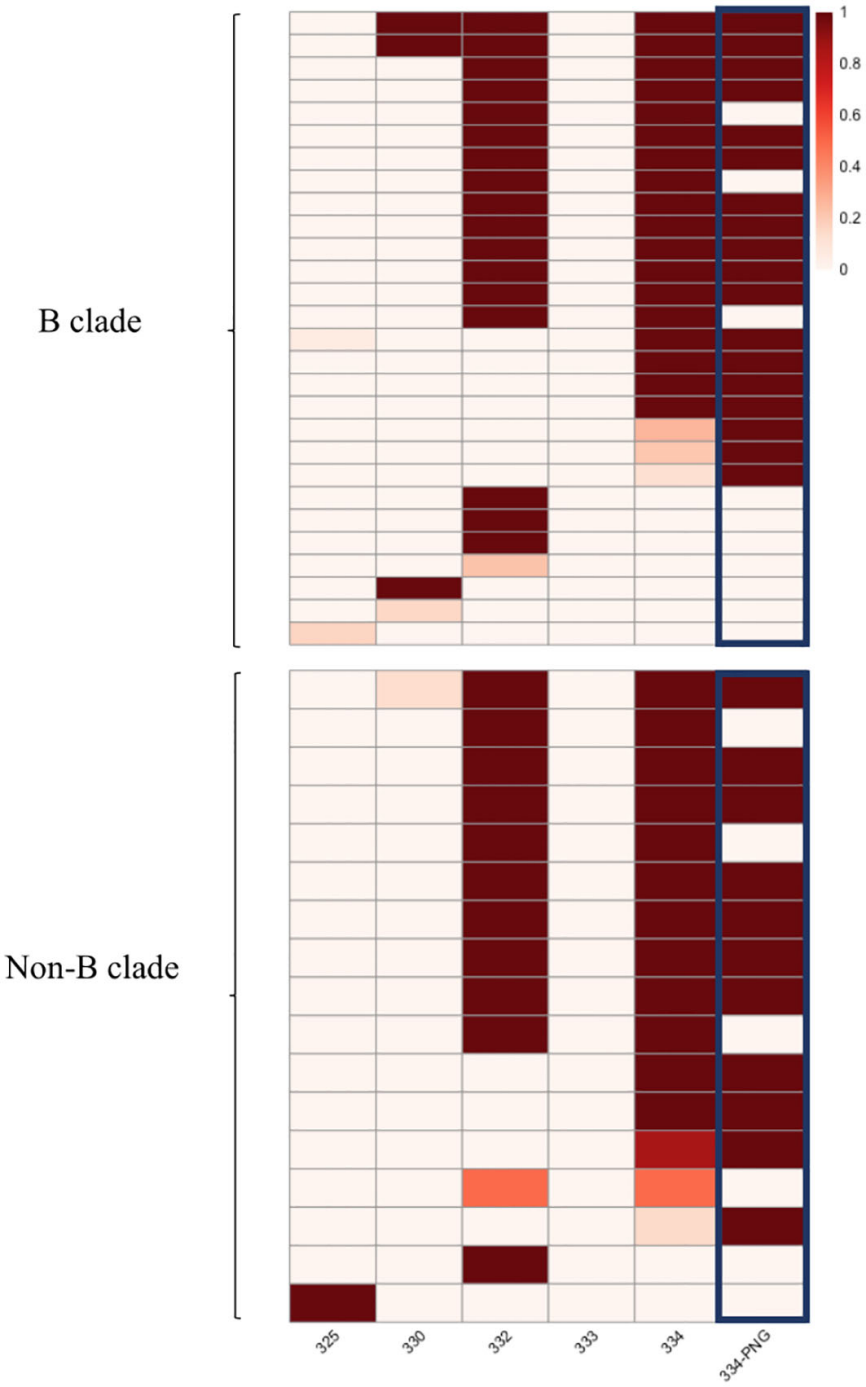
Heatmap presenting the frequency of mutated sites in the HEATHER cohort samples. This heatmap illustrates the frequency of mutations in the protein sequence in individual samples at HXB2 Env positions associated with 10-1074 susceptibility. Each row in the heatmap represents a distinct sample, and the first five columns correspond to precise amino acid positions. The last column illustrates the presence of a PNG at position 334. All samples harbouring >1 sequence with predicted resistance to 10-1074 B clade samples are shown in the upper panel and non-B are shown in the lower. The colour scale shows the percentage of sequences with 10-1074-associated mutations, per sample.

Mutations that interfere with the glycan attachment on the PNG at HIV Envelope position 334 were the most common among sequences predicted to be resistant (35 of the 45 sequences with resistance-associated mutations, 77.7%) in the HEATHER cohort. More than half of the potentially resistant sequences (25 of the 45 sequences with resistance-associated mutations, 55.5%) carried mutations at both positions 332 and 334 (**Figure 2**) and furthermore, 64.4% of likely resistant samples in the cohort had a PNG site shift from position 332 to position 334 (**Figure 2**). Notably, 89.2% of those with a PNG at 334 in this dataset also carried a 336T (^334^N-x-T^336^), which has a 100% likelihood of glycan occupancy (21). However, ^332^N-x-T^334^ was very rare in the sequences with an intact 332PNG. Furthermore, mutations at 330, which has been described as a critical 10-1074 binding site on *env*, most often occur with mutations that affect the glycan binding at position 332 (50% of 4 sequences with 330 mutations in the HEATHER cohort) (**Figure 2**).

### 10-1074 sensitivity prediction in B clade viruses over the course of the HIV epidemic

**Figure 3:**
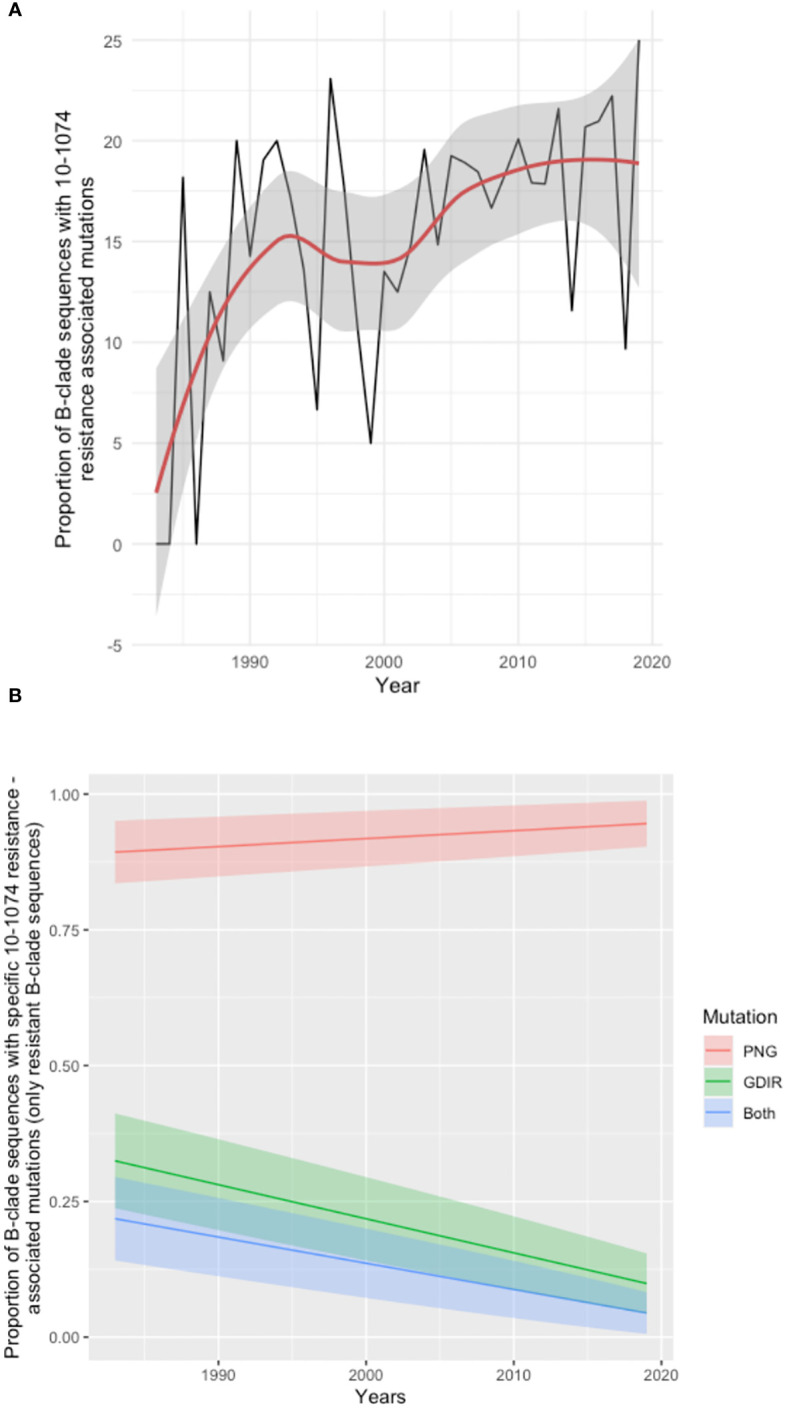
Analysis of Los Alamos Database B clade *env* sequences. **(A)** Time series analysis showing the proportion of sequences with 10-1074 resistance-associated mutations in the total number of sequences available per year. The trend line is shown in red, and the confidence intervals are shaded grey. Years for which less than 5 sequences where available were excluded from the analysis. **(B)** Frequency of individual mutation patterns associated with 10-1074 resistance among all sequences predicted as resistant, sampled throughout the pandemic. The type of mutation is encoded as ‘PNG’, for mutations affecting the 332-glycan binding (332-334 sites), ‘GDIR’ for mutations impacting the binding site on the protein (sites 325 and 330) and ‘Both’ for mutations occurring in both regions in the same sequence. Each mutation pattern is marked with a different colour. The year of sampling is on the x-axis and the proportion of resistant B-clade sequences per year is on the axis. The coloured bands represent the confidence interval for each fitted line.

To explore further the broader prevalence of resistance to 10-1074, 6516 HIV *env* protein sequences sampled between 1983 and 2019 were downloaded from the Los Alamos sequence database (dataset Year 2020, all HIV-1 subtypes included; one sequence per individual) and tested for mutations associated with resistance to 10-1074. Of these, we focused on subtype B, and any sequences for which the time of sampling was unknown (43 sequences) were excluded from the analysis. The resulting analysis of 2425 B clade HIV *env* sequences revealed a trend of increasing 10-1074 resistance over the course of the HIV pandemic (16) (**Figure 3A**). Further analysis in the subset of sequences with 10-1074 resistance-associated mutations, reveals that mutations impacting the 332PNG (in positions 332 and/or 334) are becoming more frequent with time (**Figure 3B**), potentially driving the 10-1074 resistance pattern identified.

### Variability in hypervariable variable loop length between resistant and sensitive B clade sequences

**Figure 4:**
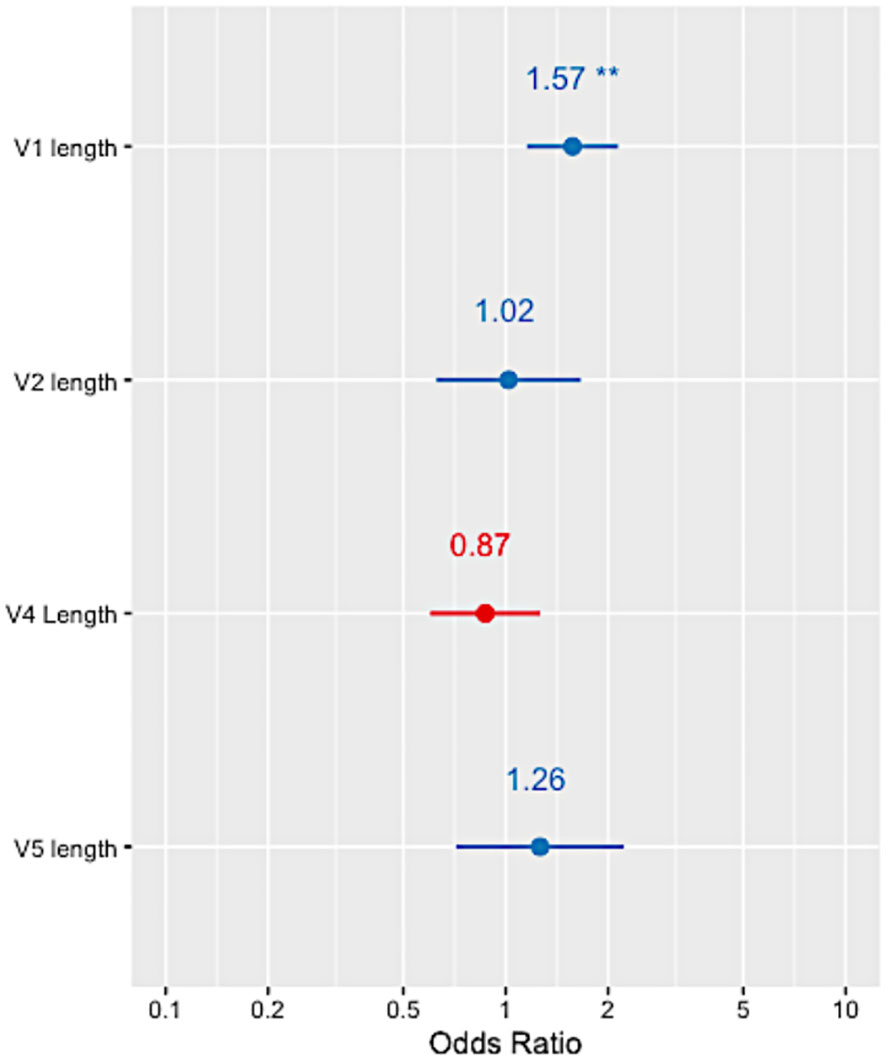
Forest plots showing the odds ratio of 10-1074 resistance in relation to each of the HIV Env variable loop lengths in the HEATHER cohort. ** indicates a p-value<0.001.

The variable loops in HIV Env evolve to evade the host immune response by incorporating insertions and deletions which have been associated with bNAb sensitivity (18). Alignment-independent methods were used on the HEATHER cohort sequences to measure the variable regions’ lengths (V1, V2, V4 and V5) as well as the number of PNGs within these regions (**Supplementary Table 2**). A multivariable logistic regression with mixed effects, accounting for intra-patient variability and clade, showed that only the length of variable V1 loop was significantly associated with the presence of resistance-conferring mutations (OR=1.57, p-value<0.01, **Figure 4**). No statistically significant relationship between 10-1074 resistance-conferring mutation and the number of PNGs within the variable regions was observed.

### Sensitivity evolution through ancestral state reconstruction

**Figure 5:**
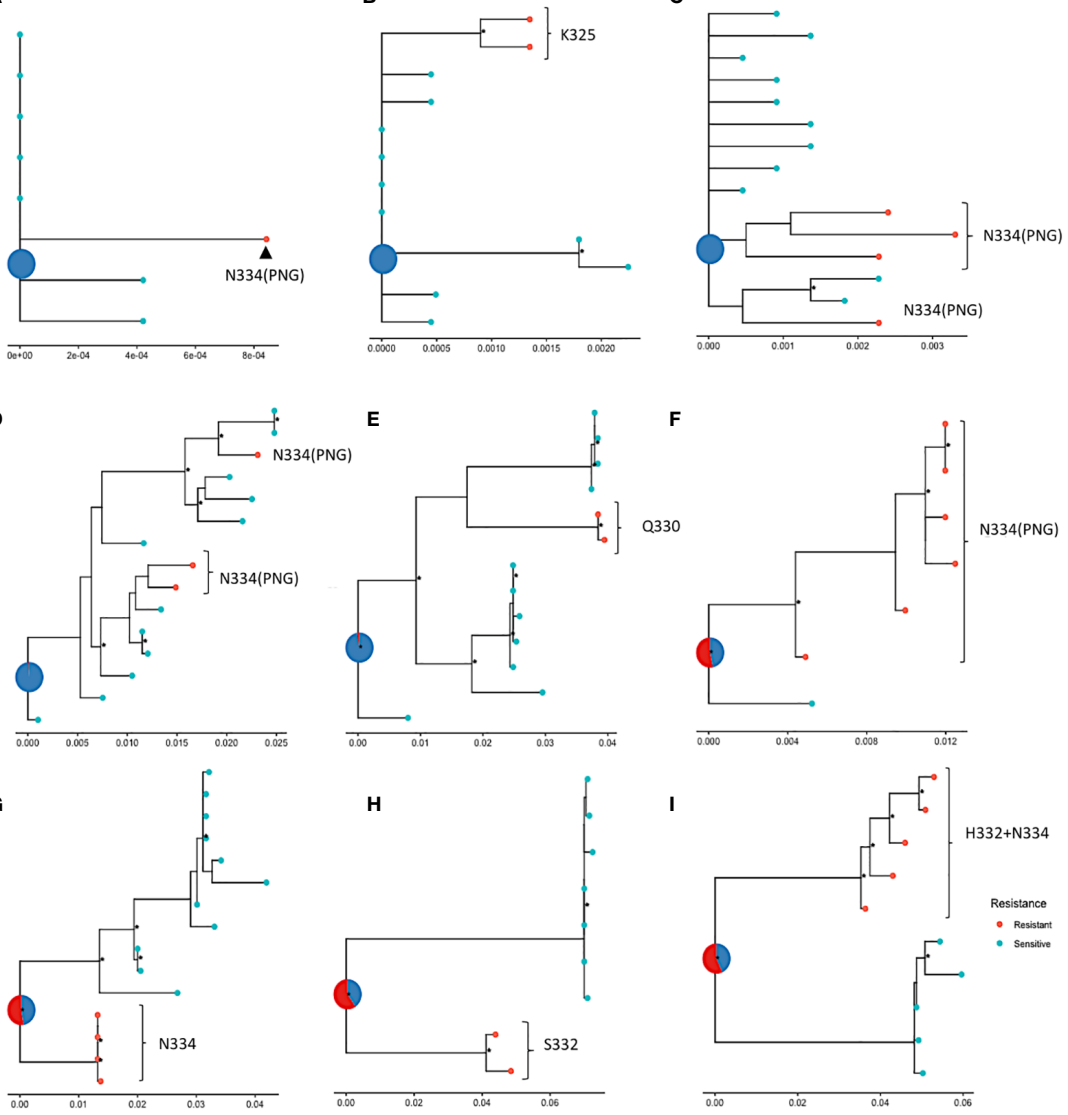
ML phylogenetic trees tracing the evolution of nucleotide sequences in nine participants **(A–I)** from the HEATHER cohort, amplified with SGA, with ancestral roots annotated with predicted sensitivity. Red points indicate 10-1074 resistant and blue points indicate sensitive sequences. The status of the most recent ancestor is represented as a pie chart at the root of the tree; **(A–E)** show trees with roots predicted to be sensitive (or most likely sensitive) to 10-1074 and **(F–I)** show trees with roots predicted to be most likely resistant to 10-1074. Data are for proviral nucleotide sequences and maximum likelihood bootstrap support values exceeding 60% are marked with a *. The scale indicating the number of mutations per site based on the length of branches can be found at the bottom of each tree plot.

In the absence of longitudinal data and relevant dating, ancestral state reconstruction can be used to infer the sensitivity phenotype of the founding virus. Here, ancestral state reconstruction was performed from the nine participants who had both sensitive and resistance sequences using bootstrapped maximum likelihood nucleotide trees. The median average pairwise distance between nucleotide sequences within samples with more than 1 sequence was 0.009 substitutions per site (range: 0-0.098 substitutions per site). The ancestral state is displayed as a pie chart at the root of each tree and the likelihood of sensitivity and resistance is indicated by different colours. The analysis revealed that in five of the samples (**Figures 5A-E**), the inferred root was very likely sensitive, indicating that the ancestral strain in these patients was most likely susceptible to 10-1074. The most common mutations observed in these samples were N334, with a PNG emerging at 334. In the remaining four trees (**Figures 5F-I**), a large amount of uncertainty was associated with the status of the most recent common ancestor of the sequences, although these ancestral strains were slightly more likely to be resistant to 10-1074. As viral evolution is unlikely on suppressive ART, this suggests that the ancestral strain in these participants may have already possessed genetic mutations associated with 10-1074 resistance, consistent with transmission of bNAb resistant strains, or that evolution of these mutations occurred shortly after transmission before ART was started.

## Discussion

This study was the first to evaluate the prevalence of 10-1074 associated mutations in a bNAb-treatment-naïve population with PHI in the UK using viral sequencing from the HIV reservoir. In the absence of a gold standard for predicting bNAb resistance, we used specific *env* residues to define 10-1074 resistance, from samples of DNA, whilst the virus was suppressed on ART, which were selected by an algorithm based on West et al. (14), which has been previously used to assess sensitivity to 10-1074 (2, 8). Previous studies have demonstrated that the recognition of the 332 glycan is necessary for 10-1074 binding, so any disruption of the glycan site would confer resistance to this bNAb (16), whereas variations on the 10-1074 GDIR binding region on the protein are better tolerated (18). In addition, we demonstrated that 10-1074 genotypic sensitivity varies among different HIV subtypes, suggesting that treatment with 10-1074 may be less effective in people who live with non-B clades of HIV, for example, subtype CRF01-AE HIV. These findings are comparable to previous study results, showing that 88.5% of the B-clade viruses were neutralised *in vitro* by 10-1074 at an average of 80% inhibitory concentrations (4) or, that CRF01-AE viruses are extremely resistant to 10-1074 (18). However, due to the low number of participants with non-B clades, these findings may need to be confirmed through studying larger, non-B PHI clade cohorts.

The analysis of the HEATHER cohort sequences identified that the most frequently appearing substitutions in resistant sequences impact the binding to the 332-supersite glycan. Instead, the PNG site was shifted from position 332 to position 334 in most of these sequences. A glycan at this position could, however, serve as a target for other neutralising antibodies and bNAbs as well as a marker of lower viral infectivity (22, 23). In addition, Caskey et al. (4) observed some reversion of 10-1074 escape mutations (such as D/K332 and I/A334) to wild type in PWH, when 10-1074 levels started dropping in the blood conferring less selection pressure, indicating that the escape mutations may have a fitness cost.

Changes in the combined length of variable region V1 have been reported to play a role in evading humoral immune responses (18, 24, 25). We found that a longer V1 loop was strongly associated with the presence of 10-1074 resistance-conferring mutations. According to previous studies, a longer V1V2 region protects against neutralising antibodies by shielding the V3 epitopes (26). The fact that a longer V1 length is associated with 10-1074 mutations may indicate that 10-1074 resistance is developed in the mutated sequences in the process of escaping from V3-targeting NAbs. Further analysis in larger sample sizes may result in more precise estimates and in elucidating the relationship between variable loop lengths and 10-1074 resistance-conferring variants.

It was not possible to clearly map the pathway of bNAb resistance evolution in this cohort with early treated PHI. However, the characterisation of ancestral resistance phenotypes in participants with mixed resistance showed that it is possible for founder viruses to be resistant or sensitive, which highlights the heterogeneity of bNAb resistance in the population. Our findings from an ART-treated primary infection cohort may differ from those in chronic infection where there is more viral exposure to immune selection pressure. The co-existence of resistant and sensitive viruses within a host may reflect viral escape from early immune response with cross-reactivity to escape from bNAbs (23). It is also possible that mutations that confer 10-1074 resistance come at a fitness cost and a resistant virus may revert to the sensitive wild type when bNAb serum levels drop and the selection pressure reduces (4). In addition, co-infection with multiple viral strains is possible and distinct clustering of viral sequences within a single sample, such as in the ones shown in **Figures 5F–I**, may indicate that a sensitive and a resistant virus were transmitted to an individual.

Choosing the best approach for predicting bNAb resistance is contentious; there is no gold standard when it comes to which assay is the most accurate (1). A similar debate explored whether phenotyping or genotyping assays were best for predicting antiretroviral therapy drug resistance, with clinical practice having now settled on a genotypic approach which has been integrated into the standard of care (2). Key advantages of genotyping over phenotypic assays are the detection of clinically significant minority resistance and the cost and time efficiency. However, the interpretation of genotypic assays and prediction algorithms has proven to be more problematic, especially for predicting bNAb sensitivity where structural interactions between amino acids are more complex than they are for drug resistance (27, 28). We have therefore been cautious here to interpret our results as likely predicting resistance, rather than being an absolute finding, although the algorithms for 10-1074 are better than for some other bNAbs, such as 3BNC117 (15, 29, 30). To accurately predict bNAb resistance, more clinical outcome data is needed matching sequence variation to viral suppression after bNAb therapy. Nevertheless, it is likely that should bNAbs become a mainstream therapy or prophylactic treatment, a genotypic approach to screening – combined with consideration of viral subtype - will be most pragmatic. This study included only samples from people treated in the UK during PHI, who had predominantly clade B HIV. This limitation may restrict the generalizability of the findings to the broader population.

In summary, the presence of pre-existing resistance presents a challenge to the effectiveness of 10-1074 treatment in PWH, potentially diminishing its long-term utility as a treatment option. This may however be minimised with proactive screening and combination therapy approaches. Should resistance be equally prevalent for other bNAbs with therapeutic potential, this has implications for screening programmes for the wide population to ensure maximum efficacy.

## Materials and methods

### Study population

Blood samples used in this study were collected from 157 participants on ART, enrolled in the HEATHER study, a prospective observational cohort study of individuals who commenced ART (and remained on uninterrupted therapy) within 3 months of the date of HIV diagnosis during PHI. Individuals were considered to have PHI if they met any of the following criteria: HIV-1 positive antibody test within 6 months of an HIV-1 negative antibody test, HIV-1 antibody negative with positive PCR (or positive p24 Ag or viral load detectable), RITA (recent incident assay test algorithm) assay result consistent with recent infection, equivocal HIV-1 antibody test supported by a repeat test within 2 weeks showing a rising optical density or having clinical manifestations of symptomatic HIV seroconversion illness supported by antigen positivity. Participants were all adult men who have sex with men and had started ART within 6 months of seroconversion. All research was performed following relevant guidelines and regulations, and all patients gave written informed consent to participate. Recruitment to the HEATHER cohort was approved by the West Midlands-South Birmingham Research Ethics Committee (reference 14/WM/1104).

### Single genome amplification

PBMCs were isolated from blood and genomic DNA was extracted using the QIAmp DNA blood midi kit (QIAGEN). HIV *env* was amplified with single genome amplification in 96-well plates. To achieve a Poisson distribution, where a maximum of 30% wells yields a product, the appropriate dilution factor was calculated for each sample. Nested PCR with Platinum Taq (Invitrogen) was used to amplify *env*, with 2 sets of primers: envB5out 5′-TAGAGCCCTGGAAGCATCCAGGAAG-3′ and envB3out 5′-TTGCTACTTGTGATTGCTCCATGT-3′ in the first round and envB5in 5′-CACCTTAGGCATCTCCTATGGCAGGAAGAAG-3′ and envB3in 5′-GTCTCGAGATACTGCTCCCACCC-3′ in the second round. If no product was amplified, an alternative set of outer primers (R3B6R 5′- TGAAGCACTCAAGGCAAGCTTTATTGAGGC-3′ and B3F3 5′-TGGAAAGGTGAAGGGGCAGTAGTAATAC-3′) was used in the first round. The first round PCR was run at 94°C for 2 min; 94°C for 15 s, 58.5°C for 30 s, and 68°C for 3 min × 35; and 68°C for 15 min. For the second round, 1 μl of the first-round product was used as a template and the mix was run at 94°C for 2 min; 94°C for 15 s, 61°C for 30 s, and 68°C for 3 min × 45; and 68°C for 15 min. The end products were run on 1% 96-well E-gels (Invitrogen) in a 1:5 dilution. Amplicons were then pooled in a sequencing library and were sequenced using MiSeq Nano kits V3 (Illumina). On average 20 sequences (range: 2-76) were sampled from each participant.

### HIV reconstruction and bNAb sensitivity prediction

A custom bioinformatics pipeline was used to assemble raw sequences and evaluate their sensitivity to 10-1074, as described elsewhere (8). Briefly, non-HIV sequences were removed, the raw sequences were aligned to HXB2, and the consensus was used as a reference to re-align raw reads. Viral sequences were translated to proteins and functionality was assessed based on *env* length and premature stop codons. Resistance to 10-1074 was defined by the absence of the following amino acids: N332; 333: not P; S/T334; D/N/T325 and H/Y330. These critical residues were associated with 10-1074 resistance using an adapted model by West et al. (14). HIV subtyping was done with REGA HIV Subtyping Tool version 3.46 (31).

### Prediction of 10-1074 sensitivity in B clade viruses over the course of the epidemic

The time series analysis to model the frequency of 10-1074 resistance-associated mutations was done and visualised using R. To model the frequency of individual mutation patterns associated with 10-1074 resistance, the subset of sequences with 10-1074 resistance-associated mutations was resampled 1000 times to account for the small dataset size and the proportion of each mutation pattern, PNG, GDIR and Both, were calculated per year for all datasets. Linear models of the proportion of each mutation pattern as a function of Year were fitted for all datasets and an average intercept and Year coefficient value were calculated. Using this data, an average line and its confidence intervals were plotted to illustrate the mutation pattern frequency trend in time.

### Variable loop features

Multiple alignment of the Los Alamos B clade sequences and of the HEATHER cohort B-clade sequences was performed with mafft (version 7.490). The lengths of variable loops as well as the number of PNGs within these regions in the aligned amino acid *env* sequences were measured using the variable region characteristics tool on the Los Alamos HIV database (https://www.hiv.lanl.gov/content/sequence/VAR_REG_CHAR/index.html). A mixed-effects logistic regression was then performed to identify the effect size of V1, V2, V4 and V5 lengths on 10-1074 resistance. The length of V3 was found to be constant across the HEATHER cohort sequences with a very small variability (1-3 amino acids compared to the average length) in very few sequences. Taking these observations, as well as similar findings in the literature (32) into account, the length of V3 was not included in the explanatory variables.

### Phylogenetic trees and ancestral reconstruction

Neighbour-joining (NJ) phylogenetic trees of all protein sequences in the HEATHER cohort were constructed using R package ape to check for inter-sample contamination. Maximum likelihood trees were built for each sample with both sensitive and resistant nucleotide sequences, using Fastree on NGphylogeny.fr. The trees were rooted on an outgroup sequence, which was then pruned while maintaining the tree structure and the position of the root. Ancestral state reconstruction was performed to estimate sensitivity to 10-1074 of the internal nodes and root. Ancestral state reconstruction was done using ape and trees were visualised using ggplot2 (33).

## Data availability statement

The data presented in this study are deposited in the GenBank repository, accession numbers PP426634-PP429225.

## Ethics statement

The studies involving humans were approved by West Midlands-South Birmingham Research Ethics Committee (reference 14/WM/1104). The studies were conducted in accordance with the local legislation and institutional requirements. The participants provided their written informed consent to participate in this study.

## Author contributions

PZ: Writing – original draft, Visualization, Methodology, Formal Analysis, Data curation, Conceptualization. ML: Investigation, Writing – review & editing. TO: Software, Writing – review & editing. JT: Writing – review & editing, Investigation. NR: Methodology, Writing – review & editing. HB: Methodology, Writing – review & editing. SK: Investigation, Writing – review & editing. PG: Supervision, Writing – review & editing. JFo: Investigation, Writing – review & editing. SF: Investigation, Funding acquisition, Conceptualization, Writing – review & editing. MA: Writing – review & editing, Supervision, Methodology, Formal Analysis. JFr: Writing – review & editing, Supervision, Investigation, Funding acquisition, Conceptualization.

## References

[1] McCoy LE McKnight Á. Lessons learned from humoral responses of HIV patients. Curr Opin HIV AIDS. (2017) 12(3):195–202. doi: 10.1097/COH.0000000000000361 28422783

[2] Mendoza P Gruell H Nogueira L Pai JA Butler AL Millard K. Combination therapy with anti-HIV-1 antibodies maintains viral suppression. Nature. (2018) 561:479–84. doi: 10.1038/s41586-018-0531-2 PMC6166473 30258136

[3] Caskey M Klein F Lorenzi JCC Seaman MS West AP Buckley N. Viraemia suppressed in HIV-1-infected humans by broadly neutralizing antibody 3BNC117. Nature. (2015) 522:487–91. doi: 10.1038/nature14411 PMC4890714 25855300

[4] Caskey M Schoofs T Gruell H Settler A Karagounis T Kreider EF. Antibody 10-1074 suppresses viremia in HIV-1-infected individuals. Nat Med. (2017) 23:185–91. doi: 10.1038/nm.4268 PMC5467219 28092665

[5] Caskey M. Broadly neutralizing antibodies for the treatment and prevention of HIV infection. Curr Opin HIV AIDS. (2020) 15:49–55. doi: 10.1097/COH.0000000000000600 31764199 PMC7340121

[6] Walsh SR Seaman MS. Broadly neutralizing antibodies for HIV-1 prevention. Front Immunol. (2021) 12:712122. doi: 10.3389/fimmu.2021.712122 34354713 PMC8329589

[7] Corey L Gilbert PB Juraska M Montefiori DC Morris L Karuna ST. Two randomized trials of neutralizing antibodies to prevent HIV-1 acquisition. N Engl J Med. (2021) 384:1003–14. doi: 10.1056/NEJMoa2031738 PMC8189692 33730454

[8] Gaebler C Nogueira L Stoffel E Oliveira TY Breton G Millard KG. Prolonged viral suppression with anti-HIV-1 antibody therapy. Nature. (2022) 606:368–74. doi: 10.1038/s41586-022-04597-1 PMC9177424 35418681

[9] Sneller MC Blazkova J Justement JS Shi V Kennedy BD Gittens K. Combination anti-HIV antibodies provide sustained virological suppression. Nature. (2022) 606:375–81. doi: 10.1038/s41586-022-04797-9 PMC11059968 35650437

[10] Bar KJ Sneller MC Harrison LJ Justement JS Overton ET Petrone ME. Effect of HIV antibody VRC01 on viral rebound after treatment interruption. N Engl J Med. (2016) 375:2037–50. doi: 10.1056/NEJMoa1608243 PMC5292134 27959728

[11] Moldt B Günthard HF Workowski KA Little SJ Eron JJ Overton ET. Evaluation of HIV-1 reservoir size and broadly neutralizing antibody susceptibility in acute antiretroviral therapy-treated individuals. AIDS. (2022) 36(2):205–14. doi: 10.1097/QAD.0000000000003088 PMC12246808 34586088

[12] Julg B Stephenson KE Wagh K Tan SC Zash R Walsh S. Safety and antiviral activity of triple combination broadly neutralizing monoclonal antibody therapy against HIV-1: a phase 1 clinical trial. Nat Med. (2022) 28:1288–96. doi: 10.1038/s41591-022-01815-1 PMC9205771 35551291

[13] Clavel F Hance AJ. HIV drug resistance. N Engl J Med. (2004) 350:1023–35. doi: 10.1056/NEJMra025195 14999114

[14] West AP Jr. Scharf L Horwitz J Klein F Nussenzweig MC Bjorkman PJ. Computational analysis of anti-HIV-1 antibody neutralization panel data to identify potential functional epitope residues. Proc Natl Acad Sci U S A. (2013) 110:10598–603. doi: 10.1073/pnas.1309215110 PMC3696754 23754383

[15] Rawi R Mall R Shen C-H Farney SK Shiakolas A Zhou J. Accurate prediction for antibody resistance of clinical HIV-1 isolates. Sci Rep. (2019) 9:14696. doi: 10.1038/s41598-019-50635-w 31604961 PMC6789020

[16] Zacharopoulou P Ansari MA Frater J. A calculated risk: Evaluating HIV resistance to the broadly neutralising antibodies10-1074 and 3BNC117. Curr Opin HIV AIDS. (2022) 17(6):352–8. doi: 10.1097/COH.0000000000000764 PMC9594129 36178770

[17] Mouquet H Scharf L Euler Z Liu Y Eden C Scheid JF. Complex-type N-glycan recognition by potent broadly neutralizing HIV antibodies. Proc Natl Acad Sci U S A. (2012) 109:E3268–77. doi: 10.1073/pnas.1217207109 PMC3511153 23115339

[18] Bricault CA Yusim K Seaman MS Yoon H Theiler J Giorgi EE. HIV-1 neutralizing antibody signatures and application to epitope-targeted vaccine design. Cell Host Microbe. (2019) 25:59–72.e8. doi: 10.1016/j.chom.2018.12.001 30629920 PMC6331341

[19] Griffith SA McCoy LE. To bnAb or Not to bnAb: Defining Broadly Neutralising Antibodies Against HIV-1. Front Immunol. (2021) 12:708227. doi: 10.3389/fimmu.2021.708227 34737737 PMC8560739

[20] Martin GE Pace M Thornhill JP Phetsouphanh C Meyerowitz J Gossez M. CD32-expressing CD4 T cells are phenotypically diverse and can contain proviral HIV DNA. Front Immunol. (2018) 9:928. doi: 10.3389/fimmu.2018.00928 29780387 PMC5946760

[21] van den Kerkhof TLGM van Gils MJ Boeser-Nunnink BD Burger JA Schuitemaker H Sanders RW. Probability of N332 glycan occupancy on HIV-1 gp120 modulates sensitivity to broadly neutralizing antibodies. AIDS. (2016) 30(14):2179–84. doi: 10.1097/QAD.0000000000001177 27258397

[22] Cai H Zhang R-S Orwenyo J Giddens J Yang Q LaBranche CC. Synthetic HIV V3 glycopeptide immunogen carrying a N334 N-glycan induces glycan-dependent antibodies with promiscuous site recognition. J Med Chem. (2018) 61:10116–25. doi: 10.1021/acs.jmedchem.8b01290 PMC6283719 30384610

[23] Anthony C York T Bekker V Matten D Selhorst P Ferreria R-C. Cooperation between strain-specific and broadly neutralizing responses limited viral escape and prolonged the exposure of the broadly neutralizing epitope. J Virol. (2017) 91(18):e00828-17. doi: 10.1128/JVI.00828-17 28679760 PMC5571269

[24] Sutar J Deshpande S Mullick R Hingankar N Patel V Bhattacharya J. Geospatial HIV-1 subtype C gp120 sequence diversity and its predicted impact on broadly neutralizing antibody sensitivity. PloS One. (2021) 16:e0251969. doi: 10.1371/journal.pone.0251969 34029329 PMC8143386

[25] van Gils MJ Bunnik EM Boeser-Nunnink BD Burger JA Terlouw-Klein M Verwer N. Longer V1V2 region with increased number of potential N-linked glycosylation sites in the HIV-1 envelope glycoprotein protects against HIV-specific neutralizing antibodies. J Virol. (2011) 85:6986–95. doi: 10.1128/JVI.00268-11 PMC3126602 21593147

[26] Rusert P Krarup A Magnus C Brandenberg OF Weber J Ehlert A-K. Interaction of the gp120 V1V2 loop with a neighboring gp120 unit shields the HIV envelope trimer against cross-neutralizing antibodies. J Exp Med. (2011) 208:1419–33. doi: 10.1084/jem.20110196 PMC3135368 21646396

[27] Mayer KH Hanna GJ D’Aquila RT. Clinical use of genotypic and phenotypic drug resistance testing to monitor antiretroviral chemotherapy. Clin Infect Dis. (2001) 32:774–82. doi: 10.1086/319231 11229846

[28] Metzner KJ. Technologies for HIV-1 drug resistance testing: inventory and needs. Curr Opin HIV AIDS. (2022) 17(4):222–8. doi: 10.1097/COH.0000000000000737 35762377

[29] Hake A Pfeifer N. Prediction of HIV-1 sensitivity to broadly neutralizing antibodies shows a trend towards resistance over time. PloS Comput Biol. (2017) 13:e1005789. doi: 10.1371/journal.pcbi.1005789 29065122 PMC5669501

[30] Yu W-H Su D Torabi J Fennessey CM Shiakolas A Lynch R. Predicting the broadly neutralizing antibody susceptibility of the HIV reservoir. JCI Insight. (2019) 4:e130153. doi: 10.1172/jci.insight.130153 31484826 PMC6777915

[31] Pineda-Peña A-C Faria NR Imbrechts S Libin P Abecasis AB Deforche K. Automated subtyping of HIV-1 genetic sequences for clinical and surveillance purposes: performance evaluation of the new REGA version 3 and seven other tools. Infect Genet Evol J Mol Epidemiol Evol Genet Infect Dis. (2013) 19:337–48. doi: 10.1016/j.meegid.2013.04.032 23660484

[32] Zolla-Pazner S Cardozo T. Structure–function relationships of HIV-1 envelope sequence-variable regions refocus vaccine design. Nat Rev Immunol. (2010) 10:527–35. doi: 10.1038/nri2801 PMC3167078 20577269

[33] Wickam H. ggplot2: Elegant Graphics for Data Analysis. New York: Springer-Verlang (2016). Available at: https://ggplot2.tidyverse.org.

