# Supplementary material for: Prevalence of resistance-associated viral variants to the HIV-specific broadly neutralising antibody 10-1074 in a UK bNAb-naïve population
Source: Front Immunol. 2024 Mar 18;15:1352123. doi: 10.3389/fimmu.2024.1352123 (PMC10982389; doi:10.3389/fimmu.2024.1352123)
Supplement: Supplementary file 1 [file DataSheet_1.pdf]

Supplementary Table 1

ID	No of Sequences	Clade	Sensitivity	Average Pairwise Distance
P1	2	CRF02_AG	Sensitive	0.07246799
P2	18	B	Sensitive	0.02565683
P3	12	B	Resistant	0.00367764
P4	5	B	Sensitive	0.0048312
P5	8	B	Sensitive	0.00174622
P6	1	B/G	Sensitive	#N/A
P7	14	B	Sensitive	0.00128248
P8	5	CRF01_AE	Resistant	0.00285215
P9	16	B	Resistant	9.4587E-05
P10	7	B	Sensitive	0.00033261
P11	17	B	Sensitive	0.00578006
P12	13	B	Sensitive	0.00261932
P13	17	B	Sensitive	0.00030816
P14	10	G	Sensitive	0.00046566
P15	6	C	Sensitive	0.00995343
P16	10	B	Sensitive	0.00044626
P17	20	B	Sensitive	0.00050548
P18	15	B	Sensitive	0.00126393
P19	16	B	Sensitive	0.00191841
P20	15	B	Sensitive	0.00554354
P21	1	B	Sensitive	#N/A
P22	8	B	Mixed	0.00029104
P23	8	C	Sensitive	0
P24	15	B	Mixed	0.00197073
P25	16	B	Sensitive	0.00810536
P26	21	D/A1	Sensitive	0.01202672
P27	18	B	Sensitive	0.00100056
P28	25	B	Sensitive	0.0003473
P29	15	B	Sensitive	0.0064416
P30	16	B	Sensitive	0.03244325
P31	9	B	Sensitive	0.00276484
P32	24	B	Sensitive	0.00067487
P33	13	B	Sensitive	0.00022387
P34	13	B	Sensitive	0.00796991
P35	14	B	Sensitive	0.01573258
P36	4	CRF12_BF	Resistant	0.00058207
P37	14	B	Sensitive	0.05896114
P38	11	B	Sensitive	0.00170388
P39	17	B	Sensitive	0.00049647

P40	16	B	Sensitive	0.00090949
P41	14	B	Sensitive	0.00209482
P42	12	B	Mixed	0.01163703
P43	10	CRF06_CPX	Mixed	0.04463416
P44	9	B	Mixed	0.04724977
P45	17	D/A1	Sensitive	0.01888525
P46	9	F1	Sensitive	0.00067908
P47	3	A1/J	Resistant	0.0983702
P48	15	B	Resistant	0.07957697
P49	6	B	Sensitive	0.00067908
P50	2	B	Sensitive	0.00029104
P51	10	B	Sensitive	0.00537447
P52	14	CRF01_AE	Resistant	4.1577E-05
P53	22	B	Sensitive	0.03500025
P54	24	B	Sensitive	0.00084042
P55	34	B	Sensitive	0.00063084
P56	30	B	Sensitive	0.00394337
P57	4	A1	Resistant	0.00029104
P58	29	B	Sensitive	0.01046798
P59	16	A1	Sensitive	0.00489911
P60	33	B	Sensitive	0.00071326
P61	19	B	Sensitive	0.00263464
P62	11	CRF01_AE	Resistant	0.00051857
P63	34	B	Sensitive	0.0008923
P64	25	G	Resistant	0.00133003
P65	29	B	Sensitive	0.00121432
P66	30	B	Resistant	7.761E-05
P67	26	B	Sensitive	0.01382645
P68	31	B	Sensitive	0.00056705
P69	3	C	Sensitive	0.00019402
P70	13	F1	Sensitive	0.00075371
P71	12	F1	Sensitive	0.01261156
P72	8	B	Sensitive	0.0003638
P73	12	CRF02_AG	Resistant	0.00070113
P74	11	CRF02_AG	Sensitive	0.00010583
P75	2	B	Sensitive	0.00029104
P76	54	A1	Resistant	0.01417708
P77	13	F1	Sensitive	0.0003582
P78	11	C	Sensitive	0.01472643
P79	5	B	Resistant	0.01583236
P80	4	B	Resistant	0.00130966
P81	2	B	Resistant	0.00087311

P82	39	B	Sensitive	0.01709203
P83	3	F1	Sensitive	0.080617
P84	4	B	Resistant	0.00058207
P85	20	B	Sensitive	0.00079346
P86	45	B	Sensitive	0.00055062
P87	24	B	Resistant	0.00121265
P88	1	B	Sensitive	#N/A
P89	5	B	Resistant	0.00069849
P90	11	B	Resistant	0.00110065
P91	14	B	Sensitive	0.00029104
P92	9	B	Resistant	0.00121265
P93	29	F1	Resistant	0.00045519
P94	16	B	Sensitive	0.01036088
P95	4	CRF01_AE	Resistant	0.00014552
P96	1	B	Sensitive	#N/A
P97	27	B	Sensitive	0.00294601
P98	3	B	Resistant	0.00019402
P99	5	CRF20-BG	Resistant	0.02686263
P100	26	B/D	Sensitive	0.00143727
P101	15	B	Sensitive	0.00038805
P102	24	B	Sensitive	0.02593806
P103	17	B	Resistant	0.00432702
P104	66	B	Sensitive	0.00252368
P105	9	CRF02_AG	Sensitive	0.0002587
P106	41	B	Sensitive	0.02307974
P107	77	B	Resistant	0.00010158
P108	23	D/A1/B	Sensitive	0.02886779
P109	11	CRF06_CPX	Sensitive	0.00088898
P110	13	F1	Sensitive	0.00569386
P111	20	B	Resistant	0.01173948
P112	2	B	Sensitive	0
P113	19	B	Sensitive	0.00022174
P114	16	B	Sensitive	0.00021828
P115	7	CRF02_AG	Mixed	0.00407451
P116	27	A1	Sensitive	0.01005526
P117	15	B	Sensitive	0.00203448
P118	33	B	Sensitive	0.01303489
P119	28	B	Sensitive	0.0082645
P120	4	B	Resistant	0.05393869
P121	4	B	Sensitive	0.00014552
P122	23	B	Sensitive	0.00052686
P123	7	B	Sensitive	0.00066523

P124	16	B	Sensitive	0.00064028
P125	15	CRF01_AE	Resistant	0.01829093
P126	1	B	Sensitive	#N/A
P127	31	B	Sensitive	0.01159796
P128	14	B	Resistant	0.00080275
P129	14	B	Sensitive	0.0516701
P130	12	G	Resistant	0.0001852
P131	14	CRF47_BF	Sensitive	0.00033581
P132	13	F1	Sensitive	0.00537297
P133	3	C	Sensitive	0.0007761
P134	12	B	Sensitive	0.00145077
P135	13	B	Mixed	0.02346945
P136	10	B	Sensitive	0.00046566
P137	12	B	Sensitive	0.02065474
P138	6	D/A1	Sensitive	0.03810633
P139	16	B	Resistant	0.00263388
P140	7	B	Sensitive	0.00097012
P141	3	B	Resistant	0
P142	15	B	Sensitive	0.01928322
P143	15	B	Resistant	0.00058207
P144	14	D/A1	Mixed	0.074666
P145	13	B	Sensitive	0.00070147
P146	60	B	Resistant	0.01342742
P147	42	B	Sensitive	0.00110702
P148	31	CRF02_AG	Sensitive	0.01238312
P149	20	C	Sensitive	0.00060527
P150	19	B	Mixed	0.02398548
P151	8	F1	Sensitive	0.00145518
P152	31	CRF02_AG	Sensitive	0.02186338
P153	34	C	Sensitive	0.00072837
P154	34	B	Sensitive	0.00150602
P155	72	B	Sensitive	0.00102682
P156	9	B	Sensitive	0.00171388
P157	4	B	Sensitive	0.00116414

Supplementary Table 1: Clinical and epidemiological data for the HEATHER cohort participant

Supplementary Table 2

Variable loop	Median	Range
V1+V2	71	59-93
V3	37	33-38
V4	31	20-49

V5	13	10-25
Hypervariable V1+V2	30	19-52
Hypervariable V4	15	4-33
Hypervariable V5	7	4-19
V1+V2 PNG	6	4-9
V3 PNG	2	0-2
V4 PNG	4	2-7
V5 PNG	2	0-3
Hypervariable V1+V2 PNG	4	2-7
Hypervariable V4 PNG	2	0-5
Hypervariable V5 PNG	2	0-5

Supplementary table 2: Median and range of length of variable and hypervariable Env loops and number of PNG within the same regions.
